# Geotechnology in the analysis of forest fragments in northern Mato Grosso, Brazil

**DOI:** 10.1038/s41598-018-22311-y

**Published:** 2018-03-02

**Authors:** Sergio Guedes Barbosa, Aline Gonçalves Spletozer, Mariane Paulina Batalha Roque, José Ambrosio Ferreira Neto, Herly Carlos Teixeira Dias, Marcony Paulo Ramos, Michael Alejandro Castro Bonilla, Wellington Souto Ribeiro, Ricardo Alcántara-de la Cruz, José Cola Zanuncio

**Affiliations:** 1 Departamento de Engenharia Florestal, Universidade Federal de Viçosa, 36570-900 Viçosa, Minas Gerais Brazil; 2 Departamento de Economia Rural, Universidade Federal de Viçosa, 36570-900 Viçosa, Minas Gerais Brazil; 3 Departamento de Engenharia Civil, Universidade Federal de Viçosa, 36570-900 Viçosa, Minas Gerais Brazil; 4 Departamento de Biologia Vegetal, Universidade Federal de Viçosa, 36570-900 Viçosa, Minas Gerais Brazil; 5 Departamento de Entomologia/BIOAGRO, Universidade Federal de Viçosa, 36570-900 Viçosa, Minas Gerais Brazil

## Abstract

Pasture implantation fragments and reduces the Amazonian forest area. The objective was to quantify landscape changes in 1985, 2000 and 2015 in northern Mato Grosso, Brazil. The study was carried out in three scenes obtained by the LANDSAT satellite of a microbasin (2742.33 ha) in the municipality of Alta Floresta. Forest, water bodies, pasture and exposed soil were the thematic classes determined to e mapping the land use evolution. The edge, density and shape indexes of the fragments were measured. Normalized vegetation difference (NDVI) values were high in 1985. Land use and occupation over 15 years (1985–2000) reduced forest cover by 69.8%, but it increased by 1.7% over the next 15 years (2000–2015). The number of exposed soil patches increased between the periods, but the total area and number of the patches of the forest fragments decreased. The high values of NDVI in 1985 showed vegetated areas with high density. Reducing forest cover decreases the size of the fragments, increases the isolation and the number of soil patches exposed. The mapping of land use showed a reduction of the Amazon forest in the microbasin in the north of Mato Grosso, in the years 2000 and 2015 compared to 1985.

## Introduction

In the early 1980s, the Brazilian government encouraged deforestation of native forest by colonizers to occupy and own Amazonian lands^1^. Currently, the Brazilian legislation is focused on the conservation and restoration of this native vegetation^2^.

Forest fragments are areas of natural vegetation interrupted by anthropogenic or natural barriers, reducing the animal wild, pollen and seed flow^3^. The implantation of pastures for livestock replaces the natural landscape and fragments the southern environment of the Amazon forest in the northern region of Mato Grosso, Brazil^4^. The ecological characterization of fragments contributes to proper managing and conserving these forest remnants, including at the microbasin level^5^.

Landscape ecology can be analyzed with computational tools, especially the Geographic Information System (GIS) with image processing^6^. A set of procedures and measures, known as landscape metrics, allows quantitative understanding and estimating the landscape structure patterns^7^. GIS quantifies the particularities of the landscape^8^ and, when incorporated into Remote Sensing, analyzes the physical environment through a geo-referenced database at different dates and scales^9^.

Remote densing data are used to monitor vegetation and distinguish anthropic events^10^ Vegetation indexes are based on linear combinations of spectral data, enhancing vegetation presence^11^. The normalized difference vegetation index (NDVI) emphasizes variations of band density for environmental analysis with conclusions based on the vegetal cover dynamics^12^. NDVI has been used to classify the distribution^13^ and to study the variability of vegetation biophysical parameters such as phytomass production^14^, leaf area index^15^, land use and occupation^16^, vegetation fragmentation^17^ and estimating agricultural productivity^18^.

Using maps and satellite imagery information allows evaluating the digital classification accuracy of topics from data classified and expressed as an error matrix^19^. Kappa index is important to supervising classification confidence analysis representing all the elements of the matrix^20^. Land use and land cover maps and the fragmentation dynamics analysis are environmental monitoring and preservation mechanisms for decision-making, mainly in priority areas^21^, as well as microbasins in regions with great deforestation.

**Figure 1:**
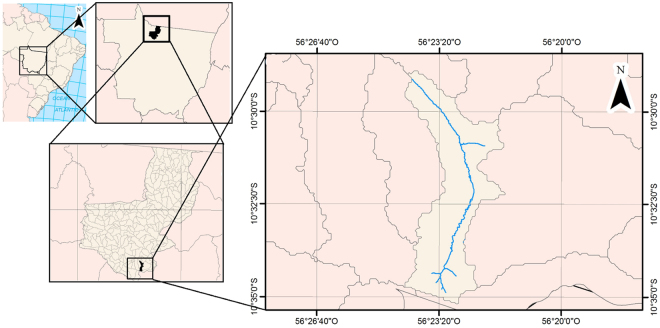
Microbasin location studied and Teles Pires river, Alta Floresta municipality, Mato Grosso State, Brazil, generated with the ArcGis 10.4 software.

The objective was to verify and to quantify structural changes of the landscape in 1985, 2000 and 2015 in a microbasin of the Teles Pires river at the Alta Floresta municipality, Mato Grosso, Brazil (Fig. 1), colonized in early 1980s, using GIS and Remote Sensing.

## Results

### Changes in land use and occupation

**Figure 2:**
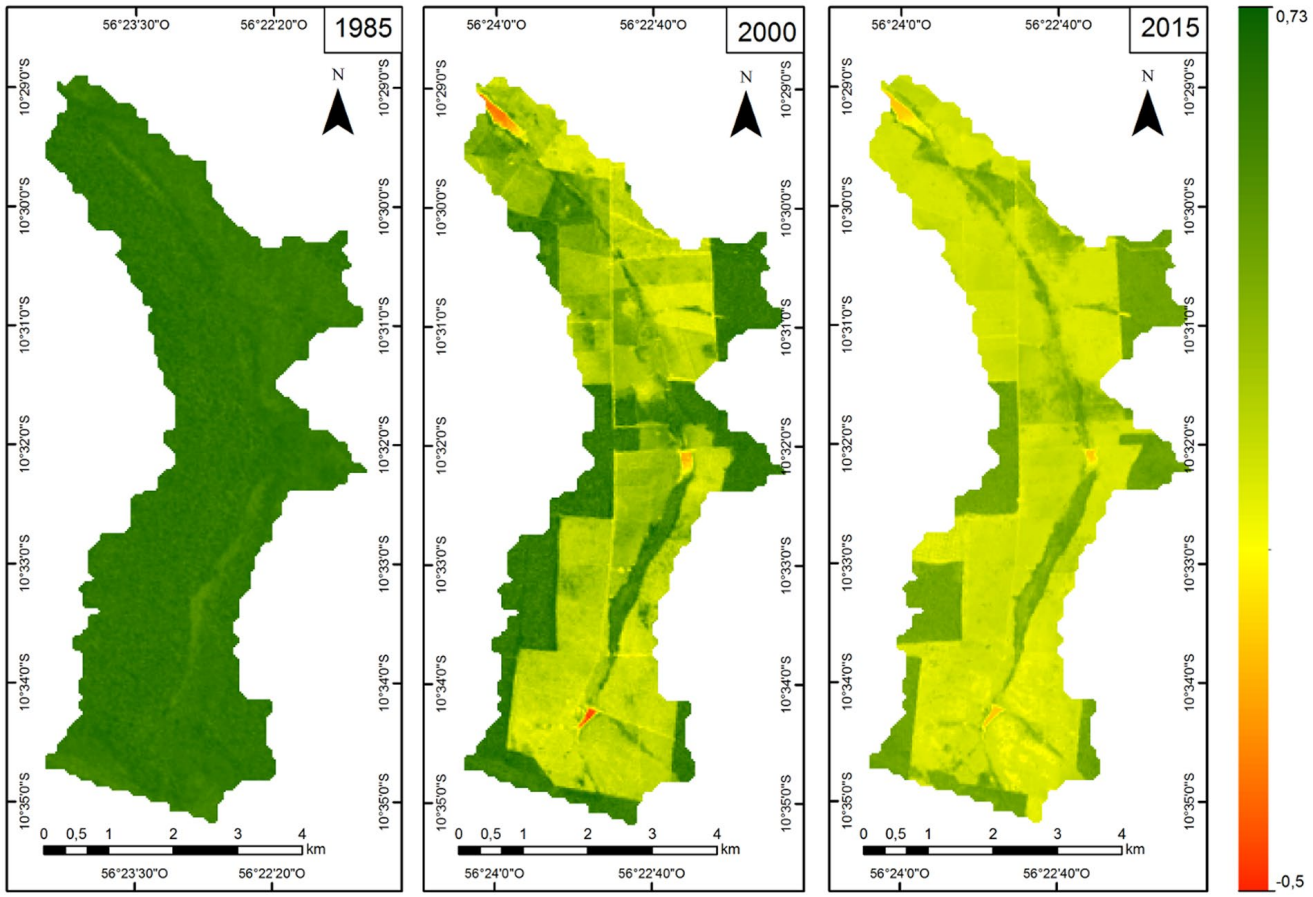
Normalized vegetation difference values (NDVI) in 1985, 2000 and 2015 for the microbasin analyzed in Alta Floresta, Mato Grosso State, Brazil, generated with the ArcGis 10.4 software.

NDVIs from the Alta Floresta microbasin ranged from 0.48 to 0.71 in 1985, −0.47 to 0.73 in 2000 and −0.06 to 0.50 in 2015. The tonality range in the Alta Floresta microbasin differed in 1985, 2000 and 2015 (Fig. 2).

Kappa indexes were 1.00, 0.94 and 0.98 in 1985, 2000 and 2015, respectively. The classification of 1985 did not present confusion among classes because only forest was detected. In 2000, the classification showed confusion between the pasture class and soil exposed with 99.2 and 96.5% of precision, respectively, but the accuracy for the other classes was 100%. The precision in 2015 was 99.7 e 99.6% for exposed soil and pasture and 100% for the other classes.

**Figure 3:**
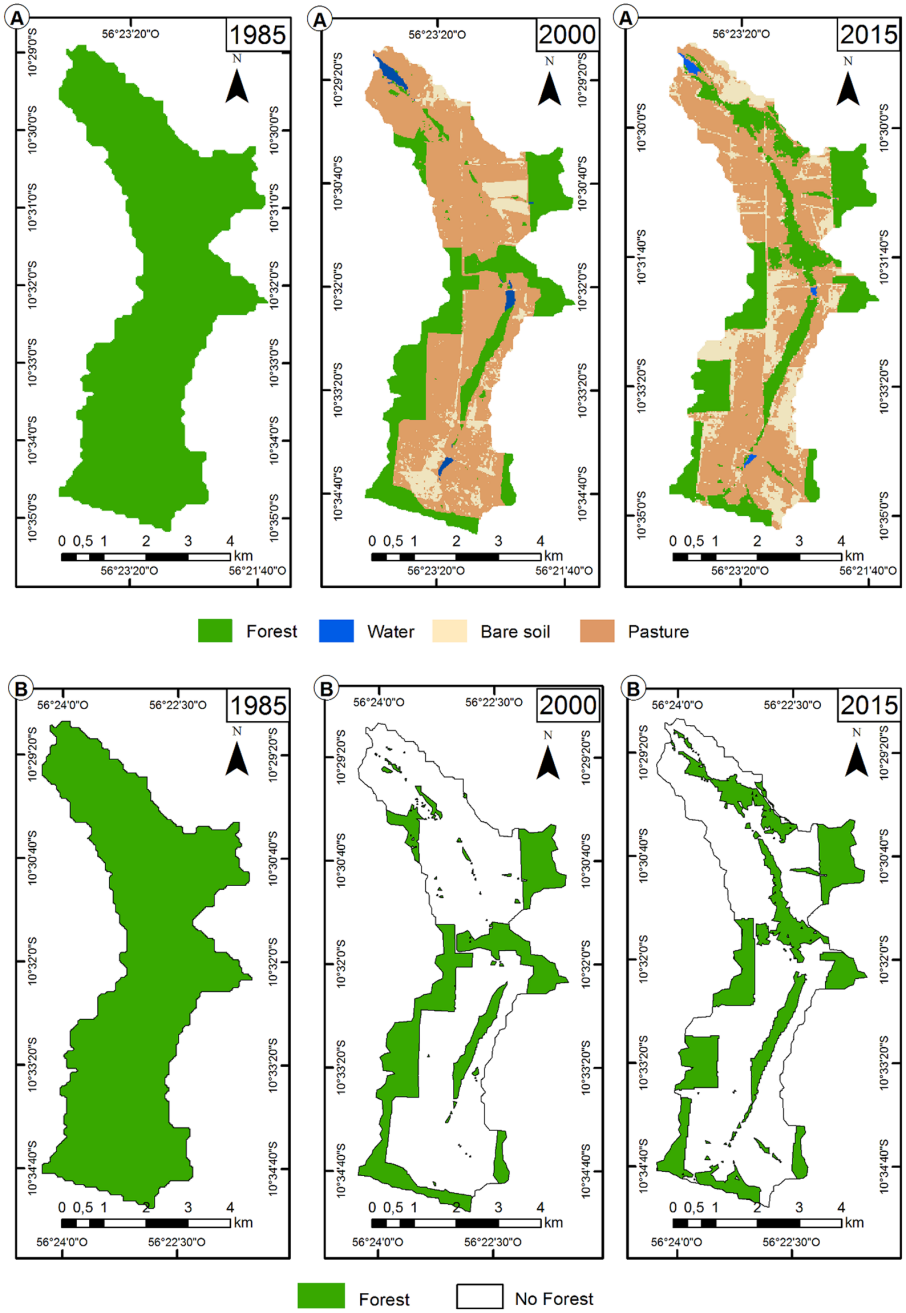
Evolution of land use classes (**A**) and forest fragments (**B**) in 1985, 2000 and 2015 of the microbasin in Alta Floresta municipality, Mato Grosso State, Brazil, generated with the ArcGis 10.4 software.

In 1985, 100% of the vegetation was native, reduced to 30% 15 years later, followed by an increase of ≈2% in 2015 (Table 1, Fig. 3A). Water bodies decreased in 27 ha between 2000 and 2015, and the soil class exposed increased from 0.0 ha in 1985 to 476.3 ha in 2015. NDWI confirmed the reduction of water bodies at the Alta Floresta municipality (data not show). Pasture area was lower in 2000 than in 2015 (Table 1).

**Table 1:** Area (hectares) of land use and occupation of classes per year in 1985, 2000 and 2015 in the microbasin studied at the Alta Floresta municipality, Mato Grosso State, Brazil.

Classes per year	1985		2000		2015		Changes Areas (ha)		
	Area	(%)	Area	(%)	Area	(%)	2000–1985	2015–1985	2015–2000
Native vegetation	2742.33	100	828.16	30.20	874.87	31.90	−1914.17	−1867.46	46.70
Pasture	0	0	1610.14	58.71	1375.63	50.16	1610.14	1375.63	−234.51
Exposed soil	0	0	261.35	9.53	476.34	17.37	261.35	476.34	214.99
Water bodies	0	0	42.68	1.56	15.49	0.56	42.68	15.49	−27.18
Total	2742.33	100	2742.33	100	2742.33	100	—	—	—

### Quantitative analysis of landscape structure

Temporal analysis of the microbasin landscape indexes (Table 2, Fig. 3B) showed an increase in the number of fragments (Nsp) from one in 1985 to 60 in 2000, and a decrease in the average size (AS) of the same from 2741.1 ha in 1985 to 13.8 ha in 2000. The increase of the Nsp from 1985 to 2000 increased the standard deviation of forest patches (SDS) and the coefficient of variation of the same spot (CoVs). The largest period from the three periods analyzed was in the 2000 with approximate values of 55.5 m² and 402.0% for SDS and CoVs, respectively.

**Table 2:** Landscape ecology indexes calculated by the Patch Analyst for 1985, 2000 and 2015 in the microbasin studied at the Alta Floresta municipality, Mato Grosso, Brazil.

Group	Indexes*	Unit	Period		
			1985	2000	2015
Area	AAs	Hectares	2741.10	828.46	874.75
Density and size	AS	Hectares	2741.10	13.81	15.08
	Nsp	Dimensionless	1	60	58
	SDS	m²	0.00	55.51	33.52
	CoVs	Porcent	0.00	402.02	222.26
Edge	BD	Meters (m)	45161.93	68071.64	85881.42
	TB	m/m²	0.00	0.01	0.01
Form	IAS	Dimensionless	2.43	1.43	1.48

^*^AAs: area of all stains; AS: average size; Nsp: Number of spots; SDS: Standard deviation of size; CoVs: Coefficient of variation of size; BD: Border density; TB Total borders; and IAS: Index and average shapeand.

Calculations of landscape metrics for the microbasin showed that the total fragment borders increased from 45161.9 m^2^ in 1985 to 68071.6 m^2^ in 2000 (intermediate), and 85881.4 m^2^ in 2015 (higher). The shape index (IAS) decreased from 2.23 to 1.43 from 1985 to 2000, respectively (Table 2).

## Discussion

The NDVI in 1985 showed that the microbasin was covered by forests due to darker shades, and the NDVI values of 0.60 are common in regions of tropical rainforest^22,23^. Negative NDVI values in 2000 and 2015 show reduced native vegetation with soil exposure and watercourses. NDVI variability highlights changes in vegetation cover that may be related to anthropic action (pastures, roads, occupations)^24^, as reduction observed in forest areas for agricultural use in Chile from 1975 to 2005^25^ and in Ecuador from 1982 to 2015^26^.

Kappa indexes higher than 0.75 validated the supervised classifications, with accuracy above 96% for all classes, and excellent concordance for the forest class^27^. The low confusion between pasture and exposed soil is related to the similarities of the spectral responses that these classes have with each other^28^. These indexes confirm that the data collected correctly represent the measured variables, and consider that the classifications are statistically correct^29^.

The reduction by 30% of the native vegetation between 1985 and 2000 was due to conversion of forest areas to pasture and exposed soil by the anthropogenic activities, being the pasture implementations the highest responsible for the reduction (58.7%). Deforestation in Chile by the implementation of forest plantations increased from 5.5% in 1975 to 42.4% in 2007, corroborating as a direct deforestation cause and biodiversity loss^30^. The analysis of the causes of these changes in the landscape allows to predict which areas are most vulnerable to changes and to prevent socioenvironmental adversities^25^. The reduction of native vegetation in Mato Grosso impacted negatively the microbasin with increased runoff, erosion and silting of rivers^31^. However, the financial and technical support, through the “Olhos D’Água da Amazônia” project for the recovery of natural areas along the watercourse, may explain the native vegetation increase between 2000 to 2015 in the Alta Floresta municipality^32^. Inadequate pasture management, such as overgrazing, compaction and soil exposure in Mato Grosso, increased over the years with a peak in 2015, similar to that reported in the vicinity of the private reserve of the national patrimony in Cafundó, Espirito Santo from 1970 to 2007^33^.

The decrease in bodies of water was due to the increase in exposed soil class^34^. Soil use and occupation results in deforestation with potential to impact processes of the hydrological cycle (precipitation, increasing surface runoff, temperature and relative humidity) which has a close relationship with evapotranspiration^35^. Water bodies decreased by 45% between 1984 and 2015 in Egypt, mainly due to increased use of land exposed by anthropogenic activities^36^. The reduction of evapotranspiration affects the climate-vegetation equilibrium by leading to a warmer and drier condition in the Amazonian ecosystems^37^. Forests sustain biodiversity, reduce soil erosion, regulate the water cycle and sequester carbon, helping to mitigate the impacts of global warming^38^.

The Nsp increase from 1985 to 2015 in Mato Grosso resulted from the typical fragmentation process, reducing the average area of fragments during the temporal scales, presenting an increment of subdivision and less connection between them^39^. The smaller fragments represent a crucial role in reducing the isolation of larger fragments^40^. However, from 2000 to 2015, we had a decrease in Nsp’s eading to increase in forest cover^41^. In 2014, there was a greater recovery of these areas, which led to a consolidation of some fragments and even its expansion, grouping other fragments.

Larger fragments as estimated by AS indicate more irregular shapes and smaller ones indicate more regular shape. Their size and shape are intrinsically bounded to the edge, that is, the smaller the fragment or more elongated, the more intense the edge effect will be, that is, reducing the inner-margin ratio^42^. It is worth noting that the more it moves away from the standard shape (the perfect circle) the more cut the shape of the spot becomes, and the more it is susceptible to the edge effect^43^. The shape of the spot is more trimmed as its pattern differs from the perfect circle being more susceptible to the edge effect^42,43^.

## Conclusions

The quantification of landscape changes in the north of Mato Grosso, in 1985, 2000 and 2015 using GIS and Remote Sensing, demonstrated structural changes in the landscape, where land occupation in 15 years (1985–2000) reduced forest cover by 69.8%, but increased by 1.7% over the next 15 years (2000–2015). The number of exposed soil patches increased from one in 1985 to 60 in 2000, and the total area of patches decreased from 2741.1 ha in 1985 to 13.8 ha in 2000. However, the border of the fragments increased from 45161.9 m² in 1985 to 68071.6 m² in 2000 and in 2015 85881.4 m² and the shapes of the fragments had their MSI decreased from 2.23 to 1.43. The mapping of land use showed a reduction of the Amazon forest in the micro basin in the north of Mato Grosso, Brazil in the years 2000 and 2015 compared to 1985.

## Material and Methods

### Characterization of the study area

The study was carried out in a microbasin with an area of 2742.3 ha in the municipality of Alta Floresta, Mato Grosso, Brazil (E 567,926 m and N 8,835,861 m, Datum SIRGAS 2000, Central Meridian −57° - Southern Hemisphere) (Fig. 1). The microbasin was classified as a low permanent preservation rate (0–25% of APP) and forest conservation (0–25% of native vegetation)^44^. The climate is Am (Köppen classification) with average temperature of 27.6 ± 2 °C and annual precipitation of 3,000 mm^45^. The Red Argisol and Lithic Neosol are predominant soils^46^ with small spots of Red Latosol. The relief compartments of the municipality correspond to the Southern Plateau of Southern Amazonia^47^. The Alta Floresta vegetation is dense and open ombrophilous, comprising the southern Amazon Basin part and savanna formations to the south of Alta Floresta^48^.

### Spatial Databases

Two scenes from the LANDSAT 5 satellite (TM/August 1985 and July 2000) and one scene from LANDSAT 8 (OLI/July 2015), both with orbit 227, point 067, spatial resolution of 30 m, and minimal coverage of clouds were used. The scenes were selected based on the visual examination, corrected at atmospheric type, converted to surface reflectance and processed by the U.S. Geological Survey (USGS) through Earth Resources Observation and Science (EROS). Microbasin and hydrography boundary vector databases were provided by the the Environment Department of the Alta Floresta municipality in Mato Grosso, Brazil. The maps were generated with the ArcGis 10.4 software^49^. A database and a project were produced with the Geocentric Reference System for the Americas (SIRGAS 2000) and Universal Transverse Merchant Projection (UTM) system, zone 21S.

### LANDSAT Image Processing

Scenes of LANDSAT 5 were georeferenced and the scenes of LANDSAT 8 were redesigned. The control points for the adequacy between the information plans were selected by georeferencing, aiming at the correct overlapping of the vector limits of the microbasin in the images. The typologies and vegetation patterns were differentiated by the normalized difference vegetation index (NDVI), identifying the water corps, pasture and soil exposed to generate a fragment typology by color difference^50^. The classes were differentiated by color differences. The NDVI, obtained with equations 1 and 2 for the LANDSAT 8 and LANDSAT 5 satellite images, respectively, was associated with vegetation density^51^. Equation 1: NDVI Landsat 8 = (Band 5) R_NIR_ − (Band 4) R_RED_/(Band 5) R_NIR_ + (Band 4) R_RED_. Equation 2: NDVI Landsat 5 = (Band 4) R_NIR_ − (Band 3) R_RED_/(Band 4) R_NIR_ + (Band 3) R_RED_, in which: R_NIR_ is the near infrared reflectance and R_RED_ is the visible red light reflectance.

### Time evolution of land use and occupation

Satellite images were classified as “supervised” in the forest cover presence or absence with the Maximum Likelihood classifier algorithm. This classification groups the patterns of similar images into land use and occupation classes and establishes classes from training samples with those of interest to the scene^52^. The thematic classes were forest, water bodies, pasture and exposed soil, defined by the supervised classification based on the visual interpretation of the false color composition, infrared and natural color. The bands RGB 543, 432, 321, corresponding to false color, infrared and natural color, respectively, were composed with the satellite LANDSAT 5 images for 1985 and 2000 and those of th e RGB 654, 543, 432, corresponding to false color, infrared and natural color, respectively, for LANDSAT 8, relative to 2015. In addition, these results were compared with those of the NDVI e Normalized difference water index (NDWI) to increase the classification reliability. Three hundred and fifty samples, polygonal of 3 × 3 pixels per period, were collected according to the four classes. The raster file generated was converted to shapefile, after classification, to obtain the class area, comprising the respective polygons sum per year. The classification quality was evaluated from the confusion matrix of the training samples collected with the Kappa index^53^.

### Quantitative analysis of forest fragments by landscape ecology indexes

The fragments were analyzed in shapefile files classified per year, corresponding to the forest vegetation class. Calculations of landscape metrics for the microbasin obtained through the Patch Analyst extension of ArcGis 10.4 software were applied to vector files generated per year without fragment size distinction. The metrics, fragment density, size, fragment shape and edge indexes were determined^54,55^.

## References

[1] Mann ML (2014). Pasture conversion and competitive cattle rents in the Amazon. Ecological economics.

[2] BRASIL, Código Florestal. Lei n° 12.651, de 25 de maio de 2012. Brasília, Diário Oficial da União http://www.planalto.gov.br/ccivil_03/_ato2011-2014/2012/lei/l12651.htm (2012).

[3] Li Y (2017). Multi-scale assessments of forest fragmentation in China. Biodiversity Science.

[4] Ruviaro CF (2016). Economic and environmental feasibility of beef production in different feed management systems in the Pampa biome, southern Brazil. Ecological Indicators.

[5] Santos AR (2016). Geotechnology and landscape ecology applied to the selection of potential forest fragments for seed harvesting. Journal of Environmental Management.

[6] Tilman D, Cassman KG, Matson PA, Naylor R, Polasky S (2002). Agricultural sustainability and intensive production practices. Nature.

[7] Thygeson AS, Skarpaas O, Stefan B, Birkemoe T, Marianne E (2017). Habitat connectivity affects specialist species richness more than generalists in veteran trees. Forest Ecology and Management.

[8] Dalloz MF, Crouzeilles R, Gomes MA, Bernardo P, Prevedello JA (2017). Metrics of landscape ecology in sig environments for analysis of forest fragments. Progress in Physical Geography.

[9] Wohlfort C, Mack B, Kuenzer C (2017). Multifaceted land cover analysis and land use change in the yellow River Basin based on dense time series Landsat: exemplary analyzes in mining, agriculture, forest and urban areas. Applied Geography.

[10] Xiao R, Wang G, Zhang Q, Zhang Z (2016). Multi-scale analysis of relationship between landscape pattern and urban river water quality in different seasons. Scientific Reports.

[11] Roumenina E, Kazandjiev V, Dimitrov P, Lukarski HD (2013). Validation of LAI and assessment of winter wheat status using spectral data and vegetation indices from spot vegetation and simulated proba-V images. Internacional Journal of Remote Sensing.

[12] Takkar AK, Desai VR, Patel A, Potdar MB (2017). Impact assessment of microbasin management programmes on land use/land cover dynamics using remote sensing and GIS. Remote Sensing Applications: Society and Environment.

[13] Zhang X, Wu S, Yan X, Chen Z (2017). 2017. A global classification of vegetation based on NDVI, rainfall and temperature. International Journal of Climatology.

[14] Costa, T. C. C., Viana, J. H. M. & Ribeiro, J. L. Semideciduous seasonal forest production of leaves and deciduousness in function of the water balance, Lai, and NDVI. *International Journal of Ecology*, **923027** (2014).

[15] Toby N, Carlson., David A (1997). On the relation between NDVI, fractional vegetation cover, and leaf area index. Remote Sensing of Environment.

[16] Deepa N, Kumar KEM, Kishore N, Krishnan G (2017). Environmental change detection using Geo- Spatial techniques in Aravalli hills and Environs (Faridabad District, Haryana). International Journal of Applied Environmental Sciences.

[17] Di Giulio M, Holderegger R, Tobias S (2009). Efeitos da fragmentação de habitat e paisagem em seres humanos e biodiversidade em paisagens densamente povoadas. Journal of Environmental Management.

[18] Cheng L (2015). Analysis of farmland fragmentation in China Modernization Demonstration Zone since “Reform and Openness”: A case study of South Jiangsu Province. Scientific Reports.

[19] Jakimow, B., Griffiths, P., Van der Linden, S., Hostert, P. Mapping pasture management in the Brazilian Amazon from dense Landsat time series. *Remote Sensing of Environment*; 0034–4257 (2017).

[20] Facco DS, Benedetti AC (2016). The temporal evolution of use and occupation of land in municipalities of the Fourth Colony – RS. Ciência e Natura.

[21] Hentz AMK, Ruza MS, Corte APD, Sanquetta CR (2014). Remote sensing techniques for estimating biomass in forest environments. Scientific Center Knowing.

[22] Khand K, Numata I, Kajaersgaard J, Vourlitis GL (2017). Dry season evapotranspiration dynamics over human-impacted landscapes in the southerm Amazon using the landsat-based metric model. Internacional Journal of Remote Sensing.

[23] Zaidi SM (2017). Landsat-5 time series analysis for land use/land cover change detection using NDVI and semi-supervised classification techniques. Polish Journal of Environmental Studies.

[24] Shabou M (2015). Soil Clay content mapping using a time series of landsat tm data in semi-arid lands. Internacional Journal of Remote Sensing.

[25] Nahuelhual L, Carmona A, Lara A, Echeverria C, González ME (2012). Land-cover change to forest plantations: proximate causes and implications for the landscape in South -central Chile. Landscape and Urban Planning.

[26] Tewksbury AP, Comber AJ, Tate NJ, Lamb A, Fisher PF (2015). A critical synthesis of remotely sensed optical image change detection techniques. Remote Sensing of Environment.

[27] Souza CHW, Mercante E, Prudente VHR, Justina DDD (2013). Methods of performance evaluation for the supervised classification of satellite imagery in determining land cover classes. Ciência e Investigacion Agrária.

[28] Hill MJ, Zhou Q, Sun Q, Schaaf CB, Palace M (2017). Relationships between vegetation indices, fractional cover retrievals and the structure and composition of Brazilian Cerrado natural vegetation. International Journal of Remote Sensing.

[29] Conger AJ (2016). Kappa and Rater Accuracy: Paradigms and Parameters. Educational and Psychological Measurement.

[30] Heilmayr R, Echeverria C, Fuentes R, Lambin EF (2016). A plantation-dominated forest transition is Chile. Applied Geography.

[31] Maselli F, Vaccarri FP, Chiesi M, D´Acqui LP (2017). Modelling and analyzing the water and carbon dynamics of mediterranean macchia by the use of ground and remote sensing data. Ecological Modelling.

[32] Secretaria Municipal de Meio Ambiente (SECMA). Projeto Olhos D’Água da Amazônia. Relatório de Avaliação de Efetividade. Alta Floresta 2016. http://www.fundoamazonia.gov.br/FundoAmazonia/export/sites/default/site_pt/Galerias/Arquivos/Relatorios_Avaliacao_Efetividade/Relatorio_Efetividade_Alta_Floresta_1.pdf (2016).

[33] Pirovani BD (2014). Spatial analysis of forest fragments in the Itapemirim River Basin, ES. Revista Árvore.

[34] Davidson EA (2012). The Amazon basin in transition. Nature.

[35] Verburg R, Filho SR, Lindoso D, Bursztyn M (2014). The impact of commodity price and conservation policy scenarios on deforestation and agricultural land use in a frontier area within the Amazon. Land Use Policy.

[36] Hossen H, Negm A (2016). Change detection in the water bodies of Burullus Lake, Northern Nile Delta, Egypt, using RS/GIS. Procedia Engineering.

[37] Silveira LGT (2017). Rainfall and deforestation recycling in the Amazon: A numerical modeling study. Brazilian Journal of Meteorology.

[38] Monkkonen M (2014). Spatially dynamic forest management to sustain biodiversity and economic returns. Journal of Environmental Management.

[39] Robinson SJB, Berg EVD, Meirelles GS, Ostle N (2015). Factors influencing early secondary succession and ecosystem carbon stocks in Brazilian Atlantic Forest. Biodiversity and Conservation.

[40] Haddad NM (2015). Habitat fragmentation and its lasting impact on Earth’s ecosystems. Science Advances.

[41] Rosa PA, Breunig FM, Almeida CM, Balbinot R (2015). Dynamic of forest fragments in the northwest of Rio Grande do Sul. Geography Teaching and Research.

[42] Silva KG, Venturin N, Carvalho WAC, Batista APB, Belan LL (2017). Spatial and temporal distribution of species diversity in semideciduous seasonal forests with occurrence of fire. Revista de Biologia Tropical.

[43] Lausch A (2015). Understanding and quantifying landscape structure – A review on relevant process characteristics, data models and landscape metrics. Ecological Modelling.

[44] Campagnolo K, Silveira GL, Miola AC, Silva RLL (2017). Permanent preservation area of a river and analysis of protection legislation of native vegetation. Ciência Florestal.

[45] Alvares CA, Stape JL, Sentelhas PC, Gonçalves JLM, Sparovek G (2013). 2014 Köppen’sclimate classification map for Brazil. Meteorologische Zeitschrift.

[46] Araujo JC, Neto MVB, Silva CB, Araujo MSB, Menezes JB (2013). Semi-detailed survey of soils of the Natuba River Basin, Pernambuco. Revista Brasileira de Geografia Física.

[47] Secretary of State for Planning and General Coordination (SEPLAN). Socio-ecological economic zoning of the State of Mato Grosso. Soil map of the state of Mato Grosso. http://www.seplan.mt.gov.br/seplandownloads (SEPLAN, 2001).

[48] Brazilian Institute of Geography and Statistics (IBGE). Technical Manual of the Brazilian vegetation. https://biblioteca.ibge.gov.br/visualizacao/livros/liv63011 (IBGE, 2012).

[49] Esri. ArcGis advanced: realease 10. 4. Redlands, CA: *Environmental Systems Research Institut*e (2016).

[50] Martins AL, Cristiano RC, Vinícius MRP, Danelichen VHM, Machado NG (2015). Changes in biophysical índices due to the chenge of land cover in native cerrado área in Mato Grosso state. Ciência e Natura.

[51] Silva AM, Silva RM, Silva BB (2015). Determination of surface temperature and estimation of the balance of radiation and evapotranspiration using landsat images and observed data. Brazilian Journal of Cartography.

[52] Carvalho EV (2017). Characterization of burned areas in the State of Tocantins in the year 2014. Forest.

[53] Perring MP (2015). Advances in restoration ecology: rising to the challenges of the coming decades. Ecology.

[54] Di Orio AP, Callas R, Schaefer RJ (2005). Forty-eight year decline and fragmentation of aspen (Populus tremuloides) in the South Warner Mountains of California. Forest Ecology and Management.

[55] Kennedy RE (2014). Trazendo uma visão ecológica da mudança para a detecção remota baseada em Landsat. Frontiers in Ecology and the Environment.

